# SDF1-CXCR4 Signaling Maintains Central Post-Stroke Pain through Mediation of Glial-Neuronal Interactions

**DOI:** 10.3389/fnmol.2017.00226

**Published:** 2017-07-21

**Authors:** Fei Yang, Wen-Jun Luo, Wei Sun, Yan Wang, Jiang-Lin Wang, Fan Yang, Chun-Li Li, Na Wei, Xiao-Liang Wang, Su-Min Guan, Jun Chen

**Affiliations:** ^1^Institute for Biomedical Sciences of Pain, Tangdu Hospital, The Fourth Military Medical University Xi’an, China; ^2^Key Laboratory of Brain Stress and Behavior, People’s Liberation Army (PLA) Xi’an, China; ^3^School of Stomatology, The Fourth Military Medical University Xi’an, China; ^4^Beijing Institute for Brain Disorders Beijing, China

**Keywords:** central post-stroke pain, intra-thalamic injection, mechanical pain hypersensitivity, SDF1-CXCR4 signaling, hypoxia inducible factor 1α, glial-neuronal crosstalk

## Abstract

Central post-stroke pain (CPSP) is an intractable central neuropathic pain that has been poorly studied mechanistically. Here we showed that stromal cell-derived factor 1 (SDF1 or CXCL12), a member of the CXC chemokine family, and its receptor CXCR4 played a key role in the development and maintenance of thalamic hemorrhagic CPSP through hypoxia inducible factor 1α (HIF-1α) mediated microglial-astrocytic-neuronal interactions. First, both intra-thalamic collagenase (ITC) and SDF1 injections could induce CPSP that was blockable and reversible by intra-thalamic administration of both AMD3100 (a selective CXCR4 antagonist) and inhibitors of microglial or astrocytic activation. Second, long-term increased-expression of SDF1 and CXCR4 that was accompanied by activations of both microglia and astrocytes following ITC could be blocked by both AMD-3100 and YC-1, a selective inhibitor of HIF-1α. AMD-3100 could also inhibit release of proinflammatory mediators (TNFα, IL1β and IL-6). Increased-expression of HIF-1α, SDF1, CXCR4, Iba1 and GFAP proteins could be induced by both ITC and intra-thalamic CoCl_2_, an inducer of HIF-1α that was blockable by both HIF-1α inhibition and CXCR4 antagonism. Finally, inhibition of HIF-1α was only effective in prevention, but not in treatment of ITC-induced CPSP. Taken together, the present study demonstrated that in the initial process of thalamic hemorrhagic state HIF-1α up-regulated SDF1-CXCR4 signaling, while in the late process SDF1-CXCR4 signaling-mediated positive feedback plays more important role in glial-glial and glial-neuronal interactions and might be a novel promising molecular target for treatment of CPSP in clinic.

## Introduction

Stroke is the leading cause of adult disability worldwide (Feigin et al., 2014). In both developed countries and high income developing countries such as China, acute treatment of stroke has been improved substantially, resulting in decrease in mortality and increase in the proportion of survivors with disability (Feigin et al., 2014; Yang et al., 2014; Ferro et al., 2016; Yang Y. et al., 2016). Central post-stroke pain (CPSP) is one of the most troublesome sequelae of both ischemic and hemorrhagic stroke caused by primary lesions affecting the central somatosensory system (Yang et al., 2014; Hosomi et al., 2015; Yang Y. et al., 2016). About 11%–32% of thalamic stroke survivors experience persistent spontaneous pain, hyperalgesia, allodynia and dysesthesia and about 50%–60% patients with CPSP are resistant to the existing analgesic therapies (Chung et al., 1996; Nasreddine and Saver, 1997). So far, the underlying mechanisms of CPSP remain largely unknown due to lack of valid animal studies (De Vloo et al., 2017). In a critical review on the animal models for CPSP, the experimental thalamic hemorrhage model induced by intra-thalamic collagenase (ITC) injection (Wasserman and Koeberle, 2009; Yang et al., 2014; Kuan et al., 2015; Yang Y. et al., 2016) was believed to be the only one with relatively high reproducibility (De Vloo et al., 2017).

As introduced initially in rats with ITC-induced hemorrhagic CPSP (Wasserman and Koeberle, 2009), activation of microglial cells and astrocytes (glial barrier) surrounding the edge of the hematoma was identified on the first day and reached peak on the seventh day after ITC. Post-treatment with systemic minocycline, a microglial inhibitor, has been shown to attenuate ITC-induced mechanical allodynia and thermal hyperalgesia, indicating that the activated microglial cells contribute to the maintenance of CPSP (Hanada et al., 2014). However, what kind of chemical substances (signaling pathways) are involved in mediation of glial-glial and glial-neuronal interactions (crosstalk) remains unknown and requires to be addressed.

Stromal cell-derived factor 1 (SDF1, also named as CXCL12), a member of the CXC chemokine family, is constitutively expressed in various kinds of cells in the peripheral and central nervous system (Reaux-Le Goazigo et al., 2012; Réaux-Le Goazigo et al., 2013). Under pathological states, activated glial cells are the main source of SDF1. In LPS-induced systemic inflammation model and bone cancer pain model, SDF1 was predominantly released by activated astrocytes in the spinal cord (Shen et al., 2014; Hang et al., 2016; Yang L. et al., 2016). Microglia was also a main source of SDF1 production in the spinal cord of rats with spared nerve injury or ischemia-reperfusion injury (Bai et al., 2016; Li et al., 2016). SDF1 exerts multiple biological functions by binding to CXCR4 (Reaux-Le Goazigo et al., 2012). Recently, several studies reported that systemic treatment with AMD3100, a highly selective CXCR4 antagonist, significantly improved the functional outcome following experimental stroke through attenuating the inflammatory response and microglial activation (Huang et al., 2013; Ruscher et al., 2013; Walter et al., 2015). Importantly, the pro-algesic effect of SDF1-CXCR4 signaling has also been verified in several pathological pain models (Oh et al., 2001; Bhangoo S. et al., 2007; Bhangoo S. K. et al., 2007; Bhangoo et al., 2009; Knerlich-Lukoschus et al., 2011; Wilson et al., 2011; Menichella et al., 2014). In a rat model of bone cancer pain or ischemia-reperfusion induced inflammatory pain, both SDF1 neutralizing antibody and AMD3100 could suppress the development of pain hypersensitivity through inhibiting astrocytic and microglial activation (Shen et al., 2014; Hu et al., 2015; Li et al., 2016). In a neuropathic pain model induced by partial sciatic nerve ligation, it has been demonstrated that SDF1-CXCR4 signaling is involved in the induction of neuropathic pain through mediating microglia-astrocyte crosstalk (Luo et al., 2016). Our recent studies have also demonstrated that SDF1-CXCR4 signaling plays critical roles in mediating the satellite glial cell-neuronal crosstalk in the dorsal root ganglion (DRG) that contributes to the hyperexcitability of primary nociceptors and transition from acute to chronic processes of inflammatory pain (Dubovy et al., 2010; Yang et al., 2015, 2017). Hypoxia inducible factor 1α (HIF-1α), a transcription factor which is closely related to the expression of SDF1 and CXCR4 under hypoxia condition, has been observed to be significantly up-regulated following ITC-induced hemorrhagic stroke (Jiang et al., 2002; Liu et al., 2010; Youn et al., 2011). Taking all these into account, we hypothesized that ITC-induced thalamic hemorrhage could induce up-regulation of SDF1-CXCR4 signaling via HIF-1α which would then maintain CPSP by neuroinflammatory microenvironment caused by microglial-astrocytic-neuronal interactions through positive feedback regulation.

## Materials and Methods

### Animals

Male Sprague-Dawley rats (280–320 g) were provided by the Laboratory Animal Center of the Fourth Military Medical University (FMMU). This study was carried out in accordance with the recommendations of the Animal Care and Use Committee of FMMU and performed in accordance with the updated Guide for the Care and Use of Laboratory Animals (8th edition, the National Academies Press, 2011). The number of rats used and their suffering were minimized. During the whole experiment, the rats were randomized.

### Surgery

Surgery was performed according to the methods described previously (Yang et al., 2014; Yang Y. et al., 2016). Rats were anesthetized with chloral hydrate (0.3 g/kg, i.p.) and placed in a stereotaxic apparatus (Narishige Scientific Instrument Lab, Japan). Collagenase type IV (0.025 U/0.25 μl, Sigma-Aldrich China, Shanghai) or saline (0.25 μl) was microinjected into the ventral posterior lateral nucleus (VPL) of the right thalamus (bregma −3.48 mm antero-posterior; 3.6 mm lateral to the midline, and 6.2 mm ventral to the brain surface) according to the stereotaxic coordinates (Paxinos and Watson, 2005). After each injection, the syringe remained for 5 min to prevent spread of the agent to the brain surface. Then the needle was slowly withdrawn, the skin closed using 4.0 sutures, and all rats were allowed to recover in individual cages for at least 3 days. Naïve rats were fed under the same conditions in a parallel manner.

### Mechanical Pain Sensitivity Testing

The mechanical pain sensitivity testing was performed by experimenters blinded to the experimental treatments (Yang et al., 2014; Yang Y. et al., 2016). The rats were placed on a metal mesh floor and habituated for 1 h before testing. Ascending graded von Frey filaments were applied from underneath the metal mesh floor to the plantar area of the appropriate hindpaw. Each von Frey filament was applied 10 times (once every several seconds). The bending force value of the von Frey filament that caused an appropriate 50% occurrence of paw withdrawal was expressed as the paw withdrawal mechanical threshold (PWMT, g). Baseline PWMT of all experimental rats were measured prior to the surgery and the effects of different drugs on PWMT were evaluated at the time point indicated in corresponding figures.

### Intra-Thalamic Drug Injections

Intra-thalamic drug injections were performed at 10 days after collagenase injection when CPSP was stably established or at 30 min before collagenase injection prior to CPSP induction. The rats were injected with minocycline hydrochloride (Sigma, 10 μg), fluorocitrate (Sigma, 1 nM), AMD3100 (Abcam, 7.5 or 15 μg) or YC-1 (Abcam, 0.2 mM) in a total volume of 1 μl over a period of 10 min. The doses used in the present study were based on or slightly modified from previous studies (Zhao et al., 2007; Trecki and Unterwald, 2009; Huang et al., 2014; Luo et al., 2014; Choi et al., 2015). Minocycline hydrochloride selectively inhibits microglial activation and proliferation, with no direct effect on neurons or astrocytes. Fluorocitrate, an inhibitor of astrocyte metabolism, inhibits the tricarboxylic acid cycle enzyme aconitase in astrocytes. AMD3100, which is a highly selective CXCR4 antagonist, was used to evaluate the roles of CXCR4 in the development of CPSP. YC-1, an established HIF-1α inhibitor, was applied to verify the regulatory effects of HIF-1α in the development of CPSP. A separate group of naïve rats underwent intra-thalamic injection of recombinant active SDF1 protein (Abcam, 50 ng) or CoCl_2_ (Sinopharm Chemical Reagent Co. Ltd, 100 mM), a well-known inducer of HIF-1α, following minocycline, fluorocitrate or AMD3100 injections to explore the cellular mechanisms underlying the pro-algesic effect of SDF1-CXCR4 signaling. The intra-thalamic injection methods were the same as above-mentioned and the injection locations were assessed histologically. Fluorocitrate was dissolved in 0.3% 2 M HCl in phosphate-buffered saline (PBS) and YC-1 was dissolved in 1% dimethyl sulfoxide (DMSO), whereas all other drugs were dissolved in sterile saline.

### Immunohistochemistry

As reported previously (Yang et al., 2015), the rats were anesthetized with chloral hydrate, then perfused with physiological saline, followed by 4% paraformaldehyde in 0.1 M PB solution. The brain was removed and postfixed overnight at 4°C and then immersed in 30% sucrose in 0.1 M PB. Brain tissue were cut into transverse sections (30 μm thick) on CM1900 freezing microtome (Leica, Germany). After blocking with 10% goat serum in PBS, the sections were incubated overnight at 4°C with the following primary antibodies: mouse anti-GFAP (1:500 Millipore), rabbit anti-Iba-1 (1:500, WAKO), mouse anti-NeuN (1:200, Millipore), Goat anti-CXCR4 (1:200, Abcam) and rabbit anti-c-Fos (1:500, Abcam). On the following day, Cy3- or FITC-conjugated secondary antibodies were incubated for 2–3 h at room temperature. For double immunostaining, sections were incubated with a mixture of primary antibodies overnight at 4°C, followed by a mixture of secondary antibodies. The images were examined under a laser scan confocal fluorescent microscope (Olympus FV1000, Japan).

### Western Blotting

As reported previously, the thalamus tissues around the hemorrhagic lesion sites were collected and homogenized in RIPA lysis buffer containing protease inhibitors (Applygen Technologies Inc., China; Yang et al., 2015, 2017; Yang Y. et al., 2016). Protein concentrations of the lysate were determined using a BCA protein assay kit (Thermo Scientific, Rockford, IL, USA). Equal amounts of protein were separated by 10% separation gels and then transferred to polyvinylidene difluoride membranes (Bio-Rad, Hercules, CA, USA). After blocking with 5% nonfat milk (Bio-Rad, Hercules, CA, USA) for 1 h at room temperature, the membranes were incubated with primary antibodies at 4°C overnight. The primary antibodies included mouse anti-HIF-1α (1:500, Abcam), rabbit anti-SDF1 (1:200, Abcam) and the antibodies mentioned in immunohistochemistry section. On the following day, the membranes were washed three times in PBST and then incubated with an HRP-conjugated secondary antibody (Bio-Rad) for 2 h at room temperature. The membranes were visualized with enhanced chemiluminescence solution (Alpha Innotech Corp) and the signals were captured with FluorChem FC2 (Alpha Innotech Corp). The density of specific bands was measured with a computer-assisted imaging analysis system and normalized to β-tubulin or β-actin intensity.

### ELISA Assay

The thalamus tissues around the hemorrhagic lesion sites were collected and homogenized in RIPA lysis buffer containing protease and phosphatase inhibitors (Applygen Technologies Inc.). After quantitative measurement of the total protein concentrations with a BCA protein assay kit (Thermo Scientific), the homogenized thalamus tissue were assayed for TNF-α, IL-1β and IL-6 (Westang, Shanghai, China) by ELISA. Each protein of interest was expressed as picograms per milligram of total proteins.

### Statistical Analysis

Data were analyzed using GraphPad Prism version 5 (GraphPad, San Diego, CA, USA) and all data were expressed as means ± SEM. Differences in changes of values of each group were tested using *t*-tests and one-way or two-way repeated ANOVA, followed by individual *post hoc* comparisons (Bonferroni or Tukey test). Linear relationships were assessed using Pearson’s correlation test. A level of *P* < 0.05 was accepted as significant.

## Results

### Long-Term Activation of Microglia and Astrocytes in Peri-Thalamic Lesion Sites Caused by ITC

**Figure 1:**
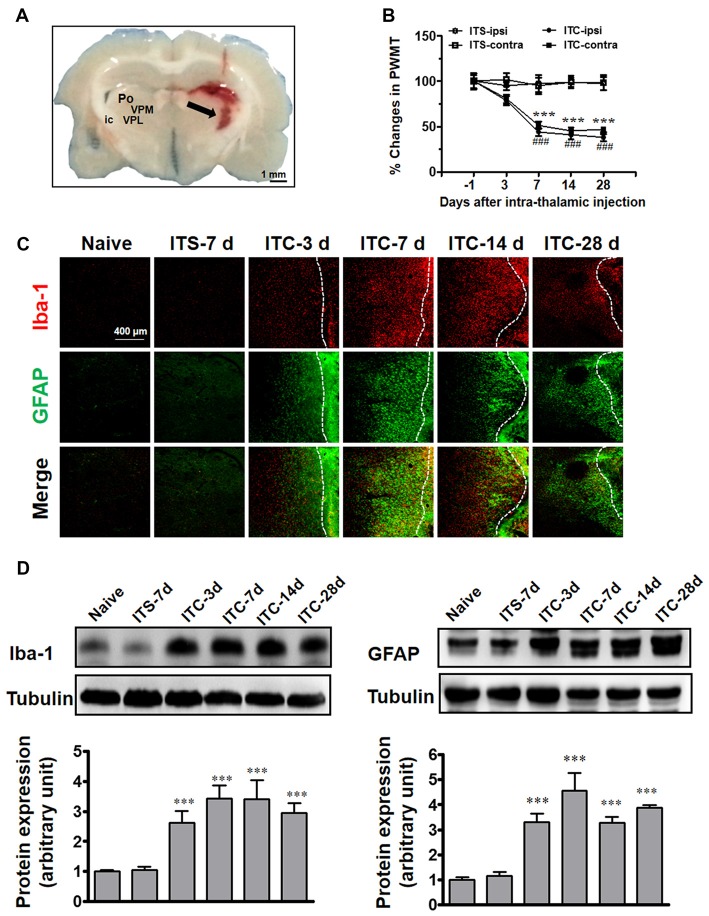
Thalamic hemorrhagic rats exhibit bilateral mechanical pain hypersensitivity and microglial and astrocytic activation in peri-thalamic lesion sites. **(A)** Photomicrograph of brain slice showing the hemorrhagic lesion site in the thalamus following ITC. Scale bar, 1 mm; ic, internal capsule; Po, posterior thalamic nuclear group; VPL, ventral posterolateral nucleus of the thalamus; VPM, ventral posteromedial nucleus of the thalamus. **(B)** Development of bilateral mechanical pain hypersensitivity induced by ITC. Saline injection served as control. ITS, intra-thalamic saline; ITC, intra-thalamic collagenase injection; contra, contralateral; ipsi, ipsilateral; PWMT, paw-withdrawal mechanical threshold; ***P < 0.001 ITC-ipsi vs. ITS-ipsi; ^###^*P* < 0.001 ITC-contra vs. ITS-contra; *n* = 10 rats/group. **(C)** Representative immunofluorescent photomicrographs showing the time course expression of Iba-1 (red) and GFAP (green), markers of microglia and astrocytes respectively, in the peri-thalamic lesion sites. The hemorrhagic lesion core is on the right side of the white line in each image. Scale bar, 400 μm. **(D)** Iba-1 and GFAP expression as examined using Western blot assay. Representative bands are shown on the top, and data summary is shown on the bottom. ***P < 0.001 vs. ITS-7 d group; *n* = 4/group.

Similar to our previous reports (Yang et al., 2014), unilateral ITC injection confined to the VPL thalamic nucleus (Figure 1A) resulted in bilateral reductions in PWMT, which were identified on day 7 post-injection and remained unchanged until day 28 post-injection, suggesting a chronic, persistent bilateral mechanical allodynia in this model (Figure 1B). To examine the involvement of microglia and astrocytes, the expression of Iba-1 and GFAP in the peri-thalamic lesion (hematoma) sites were quantified by immunohistochemistry and Western blot on 3, 7, 14 and 28 days after ITC. Compared with intra-thalamic saline (ITS) injection group, the ITC group showed marked increases in Iba-1 and GFAP expression at each time point examined. The expression level of Iba-1 and GFAP was substantially increased in peri-thalamic lesion sites on day 3 after ITC, reached peak on day 7 and remained unchanged until day 28 (Figures 1C,D). However, the expression level of Iba-1 and GFAP in the contralateral thalamus remained at basal levels (Figure 1).

### Intra-Thalamic Administration of Minocycline or Fluorocitrate Reversed ITC-Induced CPSP via Suppressing the Activation of Microglial Cells and Astrocytes

**Figure 2:**
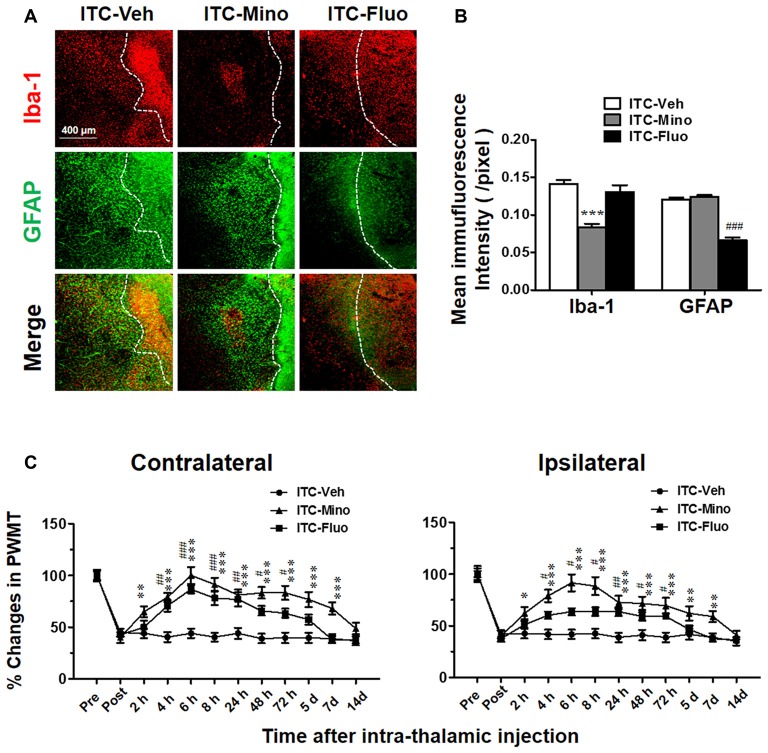
Effects of intra-thalamic injection of minocycline and fluorocitrate on Iba-1 and GFAP expression and the established central post-stroke pain (CPSP). **(A)** Representative immunofluorescent photomicrographs showing expression of Iba-1 and GFAP in peri-thalamic lesion sites following intra-thalamic minocycline (10 μg, 1 μl) and fluorocitrate (1 nM, 1μl) injection. The hemorrhagic lesion core is on the right side of the white line in each image. Scale bar, 400 μm. **(B)** Quantification of the mean immunofluorescent intensity of Iba-1 and GFAP following intra-thalamic minocycline and fluorocitrate injection. ***P < 0.001, ITC-Mino vs. ITC-Veh; ^###^*P* < 0.001, ITC-Fluo vs. ITC-Veh; *n* = 4/group. **(C)** Temporal pattern of effects of intra-thalamic minocycline and fluorocitrate injection at 10 days post thalamic hemorrhage on the established bilateral mechanical pain hypersensitivity. PWMT, paw-withdrawal mechanical threshold; *P < 0.05, **P < 0.01, ***P < 0.001, ITC-Mino vs. ITC-Veh; ^#^*P* < 0.05, ^##^*P* < 0.01, ^###^*P* < 0.001, ITC-Fluo vs. ITC-Veh; *n* = 8/group.

After CPSP was well established by 10 days after ITC, intra-thalamic injection of minocycline selectively blocked the upregulation of Iba-1 but without any effect on GFAP expression in the peri-thalamic lesion sites, while intra-thalamic injection of fluorocitrate significantly diminished ITC-induced activation of astrocytes labeled by GFAP but without any effect on Iba-1 expression (Figures 2A,B). To investigate the roles of microglial and astrocytic activation in the CPSP, we explored the time-related effects of minocycline and fluorocitrate on ITC-induced bilateral mechanical pain hypersensitivity. Our results showed that single intra-thalamic injection with minocycline alleviated the established bilateral mechanical pain hypersensitivity. The anti-allodynic effect of minocycline reached peak at 6 h after injection and maintained at a significant level for at least 7 days. The established bilateral mechanical pain hypersensitivity was also remarkably reduced by fluorocitrate, which lasted from 4 h to 3 days after injection (Figure 2C). All these results suggest that both microglia and astrocytes play important roles in maintaining the thalamic hemorrhage-induced CPSP.

### CPSP Induced by Intra-Thalamic Injection of SDF1 in Naïve Rats Was Blocked by CXCR4 Antagonism

**Figure 3:**
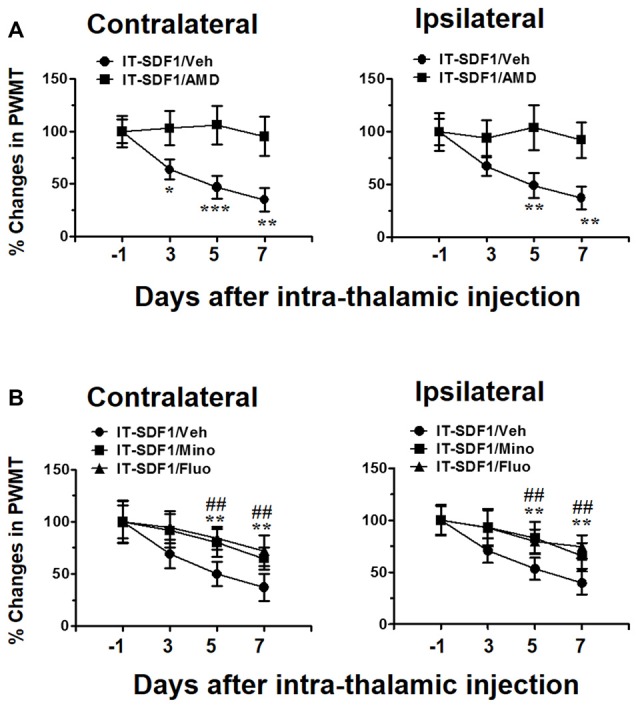
Cellular mechanisms underlying intra-thalamic stromal cell-derived factor 1 (SDF1) injection-induced mechanical pain hypersensitivity in naïve rats. **(A)** Pre-injection of AMD3100 (15 μg/μl) prevents intra-thalamic SDF1 injection-induced bilateral mechanical pain hypersensitivity. *P < 0.05, **P < 0.01, ***P < 0.001 IT-SDF1/AMD vs. IT-SDF1/Veh; *n* = 7/group. **(B)** Pre-injection of minocycline and fluorocitrate prevent intra-thalamic SDF1 protein injection-induced bilateral mechanical pain hypersensitivity. **P < 0.01 for IT-SDF1/Mino. IT-SDF1/Mino vs. IT-SDF1/Veh; ^##^*P* < 0.01 IT-SDF1/Fluo. IT-SDF1/Fluo vs. IT-SDF1/Veh, PWMT, paw-withdrawal mechanical threshold; *n* = 7/group.

As SDF1-CXCR4 signaling was closely related to microglia and astrocyte activation, we next tried to determine whether SDF1-CXCR4 signaling was involved in the induction of pain in naïve rats through regulating glial cell activation. As shown in Figure 3A, intra-thalamic SDF1 injection elicited bilateral mechanical pain hypersentivity from day 3 to day 7 which could be completely blocked by pre-administration of AMD3100 into the SDF1 injection sites. Moreover, pre-administration of minocycline or fluorocitrate in the SDF1 injection sites also significantly suppressed SDF1-induced bilateral mechanical pain hypersentivity from day 5 to day 7 (Figure 3B).

### Temporal and Spatial Changes in SDF1 and CXCR4 Expressions in the Thalamus Following ITC

**Figure 4:**
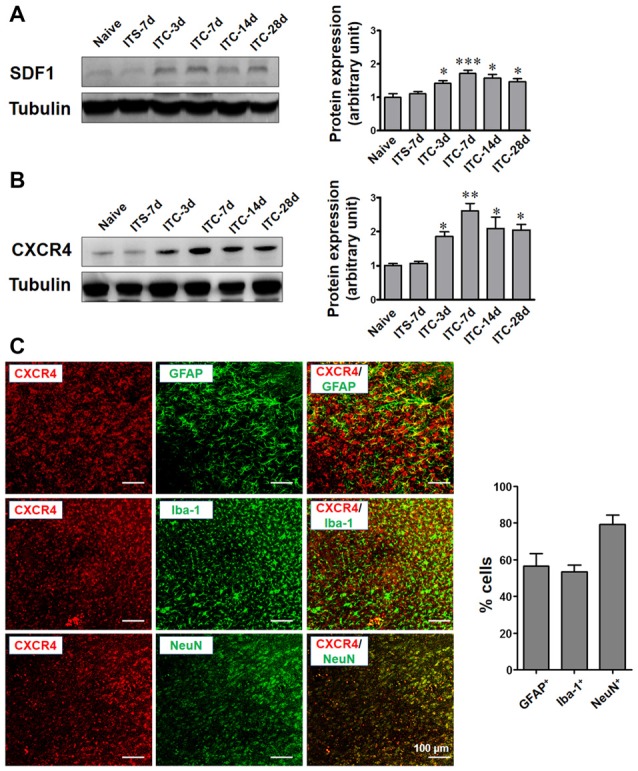
Temporal and spatial changes in SDF1 and CXCR4 expression in peri-thalamic lesion sites under CPSP condition. **(A)** Western blot assay showing the time course expression of SDF1 following thalamic hemorrhage. Representative bands are shown on the left, and data summary is shown on the right. *P < 0.05, ***P < 0.001 vs. ITS group. **(B)** Western blot assay showing the time course expression of CXCR4 following thalamic hemorrhage. Representative bands are shown on the left, and data summary is shown on the right. *P < 0.05, **P < 0.01 vs. ITS group; *n* = 4/group. **(C)** Left panel showing the immunofluorescence micrographs of the double-staining of CXCR4 with GFAP, Iba-1 and NeuN. Right panel quantifying the percentages of GFAP-positive, Iba-1-positive and NeuN-positive cells expressing CXCR4. Scale bar, 100 μm.

Under the CPSP condition induced by ITC, SDF1 expression was significantly increased in the peri-thalamic lesion sites at 3, 7, 14 and 28 days post-ITC relative to the saline control (Figure 4A). The level of CXCR4 expression in peri-thalamic lesion sites under CPSP condition was also examined by Western blot analysis. As shown in Figure 4B, increased CXCR4 protein expression was also seen at 3, 7, 14 and 28 days post-ITC relative to the saline control.

To define the cellular distribution of CXCR4, double staining of CXCR4 with different cell markers was performed. CXCR4 was not only co-localized with neuronal marker NeuN but also with astrocytic marker GFAP and microglial marker Iba-1 (Figure 4C).

### Antagonizing CXCR4 by Intra-Thalamic Administration of AMD3100 Suppressed Both Glial Cell Activation and Neuronal Hyperactivity

**Figure 5:**
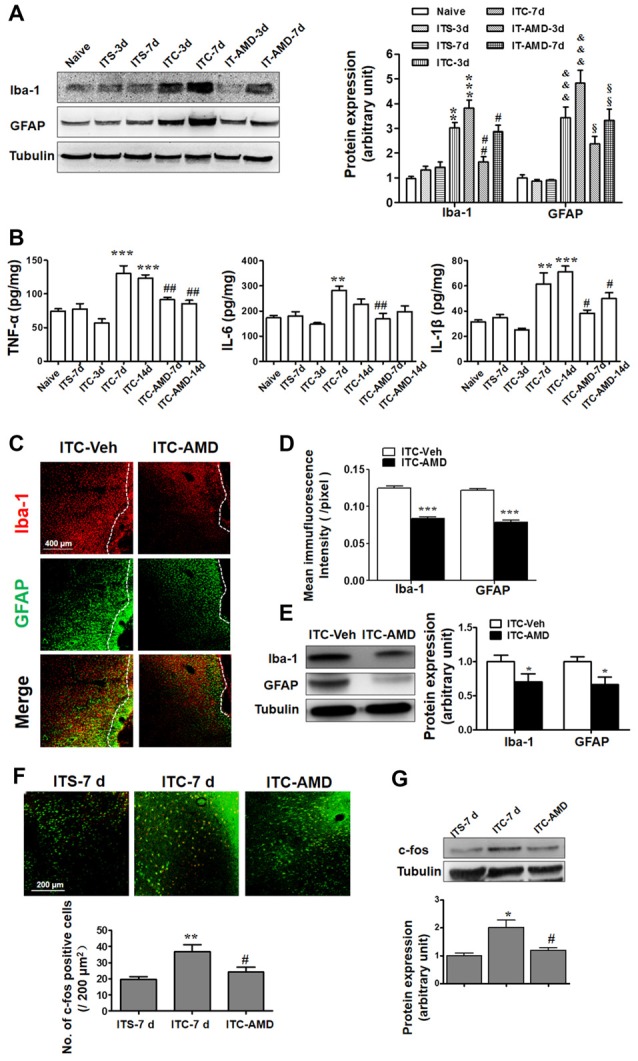
Effects of intra-thalamic injection of AMD3100 on thalamic hemorrhage-induced glial cells activation and neuronal hyperactivity. **(A)** Western blot assay showing the effect of AMD3100 (15 μg/μl), injected 30 min before ITC, on the expression level of Iba-1 and GFAP in peri-thalamic lesion sites. **P < 0.01, ***P < 0.001 vs. ITS at corresponding time points; ^&&&^*P* < 0.001 vs. ITS at corresponding time points; ^#^*P* < 0.05, ^##^*P* < 0.01 vs. ITC at corresponding time points; ^§^*P* < 0.05, ^§§^*P* < 0.01 vs. ITC at corresponding time points. **(B)** ELISA assay showing the effect of AMD3100 (15 μg/μl) pre-injection on the expression level of TNF-α, IL-6 and IL-1β. **P < 0.01, ***P < 0.001 vs. ITS-7 d; ^#^*P* < 0.05, ^##^*P* < 0.01 vs. ITC at corresponding time points. **(C)** Representative immunofluorescent photomicrographs showing expression of Iba-1 and GFAP in peri-thalamic lesion sites following intra-thalamic AMD3100 (15 μg/μl) injection at 10 days after ITC. The hemorrhagic lesion core is on the right side of the white line in each image. Scale bar, 400 μm. **(D)** Quantification of the mean immunofluorescent intensity of Iba-1 and GFAP following intra-thalamic AMD3100 (15 μg/μl) post-injection. ***P < 0.001 ITC-AMD vs. ITC-Veh; *n* = 4/group. **(E)** Western blot assay showing the effect of AMD3100 (15 μg/μl) post-injection on the expression level of Iba-1 and GFAP in peri-thalamic lesion sites. Representative bands are shown on the left, and data summary is shown on the right. *P < 0.05, vs. ITC-Veh. **(F)** Representative immunofluorescent photomicrographs showing co-expression of c-fos (red) and NenN (green) in peri-thalamic lesion sites following intra-thalamic AMD3100 (15 μg/μl) post-injection. **P < 0.01, vs. ITS-7 d; ^#^*P* < 0.05 vs. ITC-7 d;. Scale bar, 200 μm. **(G)** Western blot assay showing the effect of AMD3100 (15 μg/μl) post-injection on the expression level of c-Fos in peri-thalamic lesion sites. Representative bands are shown on the top, and data summary is shown on the bottom. *P < 0.05, vs. ITS-7 d; ^#^*P* < 0.05 vs. ITC-7 d; *n* = 3/group.

To determine whether CXCR4 contributes to microglial and astrocytic activations under CPSP condition, intra-thalamic VPL injection of AMD3100 (15 μg) was administered 30 min prior to ITC. As shown in Figure 5A, CXCR4 antagonism prevented increases in both Iba-1 and GFAP expression in the peri-thalamic lesion sites on day 3 and 7 post-ITC. Moreover, the increased release of pro-inflammatory mediators including TNF-α, IL-6 and IL-1β in the peri-thalamic lesion sites was also prevented by intra-VPL AMD3100 administration on day 7 and 14 post-ITC (Figure 5B). Furthermore, antagonizing CXCR4 by intra-thalamic AMD3100 on day 10 post-ITC also suppressed the increase in Iba-1 and GFAP expressions, implicating that SDF1-CXCR4 signaling is directly involved in the ITC-induced activations of both microglia and astrocytes (Figures 5C–E).

The role of CXCR4 in the neuronal activity was also examined by measuring c-Fos expression surrounding the glial barrier. As shown in Figure 5F, the c-Fos immunoreactivity was barely observed in rats receiving ITS, while significant increase in c-Fos-labeled neurons was observed in the peri-thalamic lesion sites following ITC. Intra-thalamic microinjection of AMD3100 reduced the number of c-Fos-labeled neurons (Figure 5F) and decreased the ITC-induced c-Fos expression as measured by Western blot (Figure 5G).

### Antagonizing CXCR4 by Intra-Thalamic Administration of AMD3100 Could Completely Reverse the Well-Established CPSP

**Figure 6:**
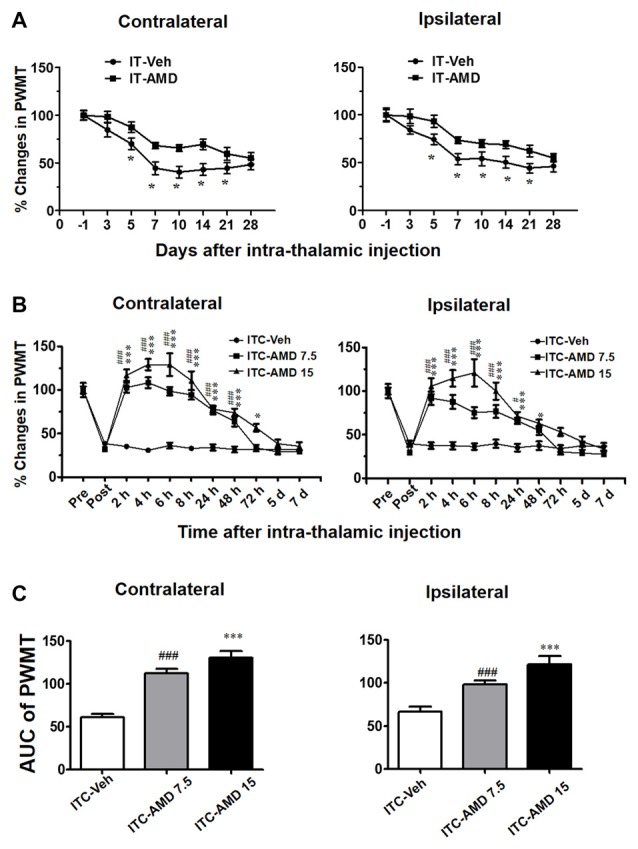
Effects of intra-thalamic AMD3100 injection on the induction and maintenance of ITC-induced CPSP. **(A)** Temporal pattern of effects of intra-thalamic AMD3100 (15 μg/μl) injection at 30 min before ITC on the induction of bilateral mechanical pain hypersensitivity. *P < 0.05, IT-AMD vs. IT-Veh; *n* = 10/group. **(B)** Temporal pattern of effects of intra-thalamic AMD3100 (7.5 and 15 μg/μl) injection at 10 days post thalamic hemorrhage on the established bilateral mechanical pain hypersensitivity. *P < 0.05, ***P < 0.001, ITC-AMD 15 vs. ITC-Veh; ^#^*P* < 0.05, ^###^*P* < 0.001, ITC-AMD 7.5 vs. ITC-Veh; *n* = 10/group. **(C)** Quantification of area under the analgesic effects of AMD3100 (7.5 and 15 μg/μl). ***P < 0.001 vs. ITC-Veh; ^###^*P* < 0.001 vs. ITC-Veh; PWMT, paw-withdrawal mechanical threshold.

To determine whether CXCR4 contributes to the induction of thalamic hemorrhage-induced CPSP, AMD3100 (15 μg) was microinjected into the VPL 30 min prior to ITC. Our behavioral assay verified that pre-injection of AMD3100 partially inhibited the ITC-induced bilateral mechanical pain hypersensitivity for 21 days relative to ITS control (Figure 6A), implying that CXCR4 plays a partial role in the initiation of CPSP.

However, the maintaining role of CXCR4 in the thalamic hemorrhage-induced CPSP was more significant. As shown in Figure 6B, intra-thalamic injection of AMD3100 with two doses (7.5 μg and 15 μg) on day 10 after ITC almost completely reversed the well-established CPSP. The analgesic effect of AMD3100 was dose-related and lasted for 48 h to 72 h, implicating the therapeutic value of AMD3100 in treatment of CPSP (Figures 6B,C).

### Inhibition of HIF-1α Blocked the Initiation but Not the Maintenance of ITC-Induced CPSP

**Figure 7:**
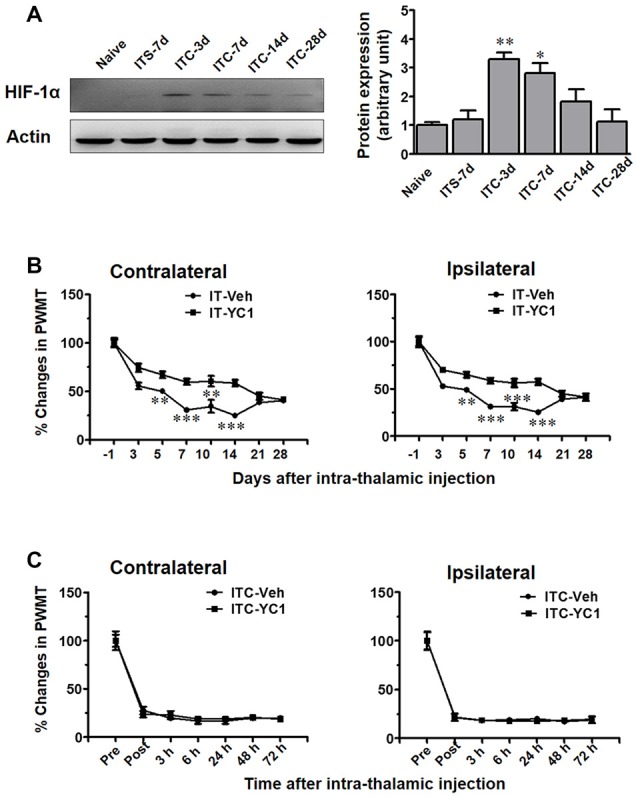
Temporal pattern of hypoxia inducible factor 1α (HIF-1α) expression in peri-thalamic lesion sites following thalamic hemorrhage and effects of intra-thalamic YC-1 injection on the induction and maintenance of CPSP. **(A)** Western blot assay showing the time course expression of HIF-1α following thalamic hemorrhage. Representative bands are shown on the left, and data summary is shown on the right. *P < 0.05, **P < 0.01 vs. ITS-7 d; *n* = 4/group. **(B)** Effect of intra-thalamic pre-injection of YC-1 (0.2 mM, 1 μl) on the induction of bilateral mechanical pain induced by thalamic hemorrhage. **P < 0.01, ***P < 0.001 IT-YC1 vs. IT-Veh; *n* = 7/group. **(C)** Effect of intra-thalamic YC-1 injection at 10 days post thalamic hemorrhage on the established bilateral mechanical pain. *n* = 7/group. PWMT, paw-withdrawal mechanical threshold.

In the present study, using Western blot analysis (Figure 7A), we examined HIF-1α expression in the peri-thalamic lesion sites at 3, 7, 14 and 28 days after ITC. It was revealed that ITC resulted in a short-term increase in HIF-1α expression, reaching peak during day 3 and 7, returning to the control level since day 14 post-ITC (Figure 7A).

We next explored whether HIF-1α is involved in the development of ITC-induced CPSP. It was interesting to find that intra-thalamic microinjection of YC-1, a potent inhibitor of HIF-1α production, blocked the occurrence of ITC-induced CPSP when being administered 30 min prior to ITC (Figure 7B), however it had no reversal effect on the well-established CPSP when being administered 10 days after ITC (Figure 7C).

### Inhibition of HIF-1α Prior to ITC Blocked the Up-Regulation of SDF1-CXCR4 Signaling and Activations of Microglia and Astrocytes

**Figure 8:**
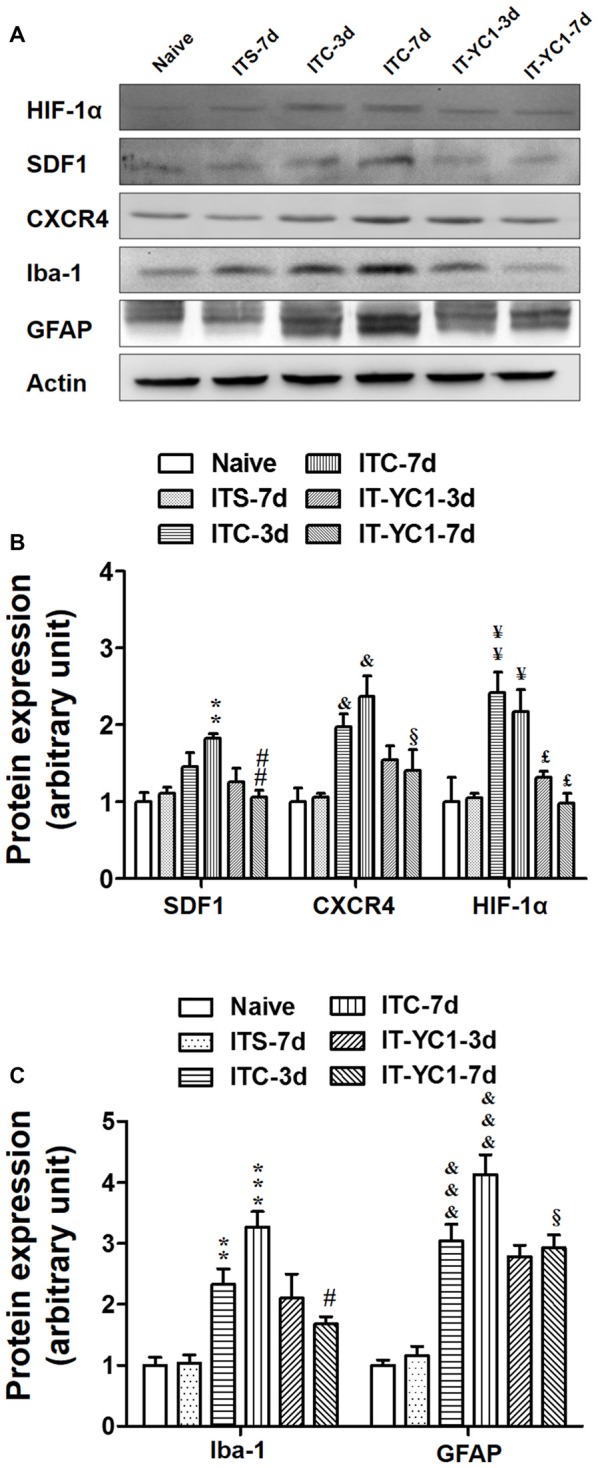
Effects of intra-thalamic pre-injection of YC-1 on the increased SDF1 and CXCR4 expression and glial cells activation induced by ITC. **(A)** Representative bands showing the effect of intra-thalamic pre-injection of YC-1 (0.2 mM, 1 μl) on thalamic hemorrhage-induced HIF-1α, SDF1 and CXCR4 up-regulation as well as on microglial marker Iba-1 and astrocytes marker GFAP up-regulation. **(B)** Quantification of Western blot assay showing that intra-thalamic pre-injection of YC-1 prevented thalamic hemorrhage-induced HIF-1α, SDF1 and CXCR4 up-regulation at 3 and 7 days. ^&^*P* < 0.05, ^¥^*P* < 0.05, ^¥¥^*P* < 0.01, **P < 0.01 vs. ITS-7 d group; ^£^*P* < 0.05, ^##^*P* < 0.01, ^§^*P* < 0.05 vs. ITC at corresponding time points; *n* = 5/group. **(C)** Quantification of Western blot assay showing that intra-thalamic pre-injection of YC-1 prevented thalamic hemorrhage-induced Iba-1 and GFAP up-regulation at 3 and 7 days. **P < 0.01, ***P < 0.001, ^&&&^*P* < 0.001 vs. ITS; ^#^*P* < 0.05, ^§^*P* < 0.05 vs. ITC at corresponding time points; *n* = 5/group.

To verify whether HIF-1α is directly involved in regulating the expression of SDF1-CXCR4 signaling under CPSP condition, intra-thalamic injection of YC-1 was administered 30 min prior to ITC. Western blot results showed that, similar to CXCR4 antagonism by AMD3100, local inhibition of HIF-1α expression by YC-1 resulted in significant reduction in the expression of both SDF1/CXCR4 (Figures 8A,B) and Iba-1/GFAP (Figures 8A,C) on day 3 and day 7 post-ITC.

**Figure 9:**
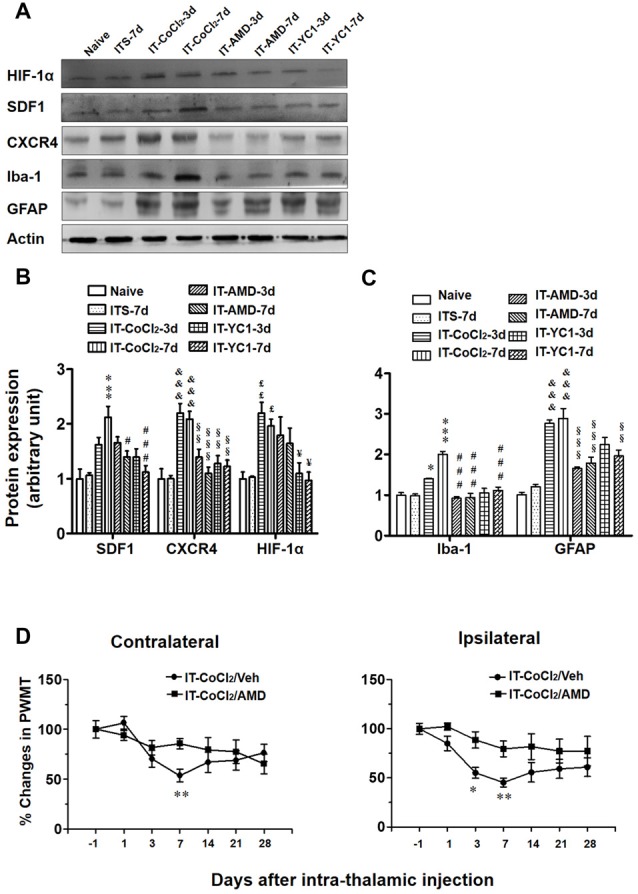
Effects of intra-thalamic injection of CoCl_2_ on HIF-1α, SDF1 and CXCR4, as well as on microglial marker Iba-1 and astrocytes marker GFAP expression in naïve rats. **(A)** Representative bands showing the effect of intra-thalamic injection of CoCl_2_ (100 mM, 1 μl) on HIF-1α, SDF1, CXCR4, as well as on Iba-1 and GFAP expression in the thalamic injection sites. **(B)** Quantification of Western blot assay showing that intra-thalamic CoCl_2_ injection increased HIF-1α, SDF1 and CXCR4 expression, and which were prevented by pre-injection of AMD3100 and YC-1. ^£^*P* < 0.05, ^££^*P* < 0.01, ^&&&^*P* < 0.001, ***P < 0.001 vs. ITS-7 d; ^§§^*P* < 0.01, ^§§§^*P* < 0.001, ^#^*P* < 0.05, ^###^*P* < 0.001, ^¥^*P* < 0.05 vs. IT-CoCl_2_ at corresponding time points; *n* = 5/group. **(C)** Quantification of Western blot assay showing that intra-thalamic CoCl_2_ injection increased Iba-1 and GFAP expression, and which were prevented by pre-injection of AMD3100 and YC-1. *P < 0.05, ***P < 0.001, ^&&&^*P* < 0.001 vs. ITS group; ^§§^*P* < 0.01, ^§§§^*P* < 0.001, ^###^*P* < 0.001, vs. IT-CoCl_2_ at corresponding time points; *n* = 5/group. **(D)** Intra-thalamic injection of CoCl_2_ induced bilateral mechanical pain hypersensitivity in naïve rats which were prevented by pre-injection of AMD3100. *P < 0.05, **P < 0.01, IT-CoCl_2_/AMD vs. IT-CoCl_2_/Veh; PWMT, paw-withdrawal mechanical threshold; *n* = 7/group.

Intra-thalamic injection of CoCl_2_, a well-known inducer of HIF-1α, was shown to result in elevation of HIF-1α, SDF1 and CXCR4 expressions examined on day 3 and 7 post-injection (Figures 9A,B). Pre-blockade of HIF-1α production by YC-1 through intra-thalamic injection prevented the enhancement of HIF-1α, SDF1 and CXCR4 expression, whereas, pre-blockade of CXCR4 with AMD3100 failed to change the enhanced expression of HIF-1α although SDF1 and CXCR4 up-regulation was prevented (Figures 9A,B). Similar to the results of ITC, intra-thalamic injection of CoCl_2_ induced overexpression of Iba-1 and GFAP (Figures 9A,C), suggesting activation of both microglia cells and astrocytes by CoCl_2_. The CoCl_2_-induced overexpression of SDF1 and CXCR4 and activations of microglia and astrocytes were also blocked by pre-injection of AMD3100 or YC-1, respectively (Figures 9A–C).

Similar to the results of ITC, intra-thalamic injection of CoCl_2_ induced bilateral mechanical pain hypersensitivity that could be blocked by pre-treatment of AMD3100 through intra-thalamic microinjection (Figure 9D).

## Discussion

**Figure 10:**
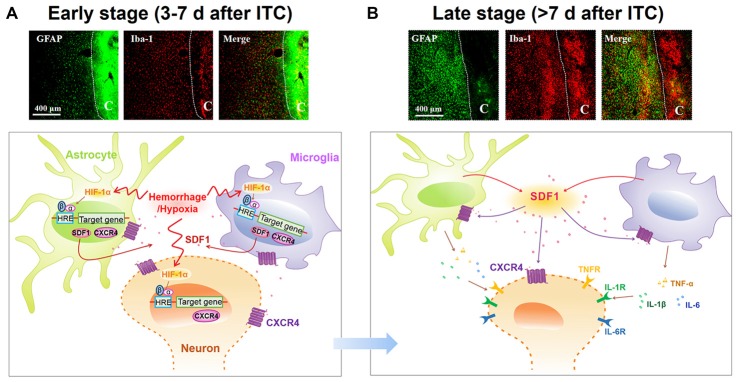
Schematic illustration of microglial-astrocytic-neuronal interactions mediated by HIF-1α-SDF1-CXCR4 signaling following thalamic hemorrhage. **(A)** The upper panel shows representative immunofluorescent photomicrographs of microglial marker Iba-1 (red) and astrocytic marker GFAP (green) expression in thalamic sections at the early stage after hemorrhage (<7 days). The hemorrhagic lesion core is on the right side of the white line in each immunofluorescent image. At this phase, hypoxia microenvironment was established in peri-thalamic lesion site which induced accumulation of HIF-1α in the cytoplasm. As a transcription factor, HIF-1α translocates into the nucleus where it dimerizes with HIF-1β subunit and forms the active HIF-1 complex which binds to the hypoxia response element (HRE) in the DNA sequence and then induces CXCR4 expression in the neurons and glial cells as well as SDF1 expression in glial cells. The synthetic CXCR4 would integrate into cell membrane forming a functional receptor for neuron and glial cells while synthetic SDF1 would be secreted into extracellular space as a ligand (lower panel). Scale bar, 400 μm. **(B)** The upper panel shows representative immunofluorescent photomicrographs of microglial marker Iba-1(red) and astrocytic marker GFAP (green) expression in thalamic sections at the late stage after hemorrhage (>7 days). The hemorrhagic lesion core is on the right side of the white line in each immunofluorescent image. At this phase, abundant SDF1 from astrocytes and microglia activates CXCR4 on glial cells, resulting in the activation of glial cells by a positive feedback activation pattern and subsequent release of proinflammatory cytokines, including TNF-α, IL-1β and IL-6, which could subsequently increase neuronal excitation and glial activation in peri-thalamic lesion site. For the survival neurons in the peri-thalamic lesion site, the released SDF1 from glial cells could bind to CXCR4 and directly evoke neuronal hyperactivity which combined with glial activation contribute to the maintenance of CPSP (lower panel). Scale bar, 400 μm.

The present study demonstrated that ITC-induced CPSP hypersensitivity was initiated by microglial-astrocytic-neuronal interactions in the peri-lesion sites through HIF-1α-dependent up-regulation of SDF1-CXCR4 signaling in microglia, astrocytes and neurons at the early stage, while it was maintained by persistent release of pro-inflammatory mediators (TNFα, IL-1β and IL-6) through SDF1-CXCR4 signaling-mediated glial-glial and glial-neuronal positive feedback regulation at the late stage. The direct involvement of SDF1-CXCR4 signaling in the induction and maintenance of CPSP was confirmed using exogenous SDF1, which was sufficient to induce mechanical pain hypersensitivity after intra-thalamic microinjection. Meanwhile, the involvement of HIF-1α in the induction, but not the maintenance, of CPSP was also confirmed by intra-thalamic YC-1 produced HIF-1α suppression and intra-thalamic CoCl_2_ produced HIF-1α over-expression. The proposed underlying mechanisms of CPSP that is mediated by microglial-astrocytic-neuronal interactions through HIF-1α-SDF1-CXCR4 signaling mediation is shown in Figure 10.

### HIF-1α Induces SDF1-CXCR4 Signaling Over-Expression and Development of CPSP

HIF-1α is a key transcription factor for the adaptive responses to hypoxia (Singh et al., 2012). In the present study, we found that the protein level of HIF-1α was significantly increased at the early phase, but not in the late phase, after hemorrhagic stroke induced by ITC. As supporting evidence, Jiang et al. (2002) reported that HIF-1α was significantly up-regulated in the basal ganglia following experimental intracerebral hemorrhage in rats receiving injections of autologous blood, thrombin, or lysed red blood cells. Here, we further found that inhibition of HIF-1α could prevent the development of CPSP but without analgesic effect during the maintenance of CPSP, implicating that up-regulation of the downstream gene expression of HIF-1α is initiated by HIF-1α but maintained by other mechanisms. By utilizing YC-1, the most specific inhibitor available for HIF-1α (Zhang et al., 2014), it was demonstrated that the increased expression of SDF1 and CXCR4 in the peri-thalamic lesion sites was almost completely suppressed. In fact, it has been demonstrated that both SDF1 and CXCR4 promoters contain hypoxia response elements (HREs) which have a HIF-1 DNA binding site (Hitchon et al., 2002; Schioppa et al., 2003; Speth et al., 2014) and blockade of HIF-1α activation can abrogate both hypoxia-induced CXCR4 up-regulation and SDF1-dependent cell migration in microglia *in vitro* (Wang et al., 2008). In the current study, the thalamic hemorrhage-induced microglial and astrocytic activations was prevented by blockade of both HIF-1α and SDF1-CXCR4 signaling, suggesting that HIF-1α plays a critical role in the up-regulation of SDF1-CXCR4 signaling that subsequently mediates the glial cell activations at the late stage of thalamic hemorrhage condition. To further verify the role of HIF-1α, we injected CoCl_2_, a well-known inducer of HIF-1α, into the VPL of normal rats. As expected, the expression of SDF1 and CXCR4 protein was up-regulated by intra-VPL CoCl_2_ microinjection and which was prevented by pre-inhibition of HIF-1α. Likewise, microglia and astrocytes was also activated by intra-VPL CoCl_2_ microinjection and the activation could be blocked by pre-treatment with CXCR4 inhibitor, indicating that HIF-1α is essential for the induction of SDF1-CXCR4 signaling-mediated glial cell activations. This result can be supported by a previous report showing that both astrocytes and microglia could be activated by microinjection of CoCl_2_ into the frontoparietal cortex of rats (Caltana et al., 2009). Most interestingly, our present study provided a new line of experimental evidence demonstrating that intra-thalamic CoCl_2_ microinjection in normal rats is sufficient to induce transient bilateral mechanical pain hypersensitivity through HIF-1α activation. HIF-1α has also been shown to be involved in the development of peripheral neuropathic pain, demonstrated by HIF-1α gene knockout in primary nociceptive neurons (Kanngiesser et al., 2014). Moreover, inhibiting accumulation of HIF-1α in the sciatic nerve also relieved both spared nerve injury-evoked and chronic constriction injury-induced neuropathic pain (Hsieh et al., 2012). However, none of these two previous studies clearly identified how HIF-1α worked to produce pain. Thus the link between up-regulation of SDF1-CXCR4 signaling by HIF-1α activation and the development of CPSP in the current study would shed new light on the treatment of CPSP in clinic.

### SDF1-CXCR4 Signaling Contributes to Maintenance of CPSP through Mediating Microglial-Astrocytic-Neuronal Interactions

To determine the roles of microglia and astrocytes in the maintenance of CPSP, we evaluated the analgesic effect of minocycline, a microglial inhibitor, and fluorocitrate, an astrocytic inhibitor, by intra-thalamic microinjection of these agents, respectively. As expected, both minocycline and fluorocitrate reversed the well-established mechanical pain hypersensitivity induced by ITC-induced hemorrhage, indicating involvement of activated microglial and astrocytic cells in the maintenance of CPSP. This result can be supported by a previous study showing attenuation of thalamic hemorrhage-induced mechanical allodynia following post-treatment with systemic minocycline (Hanada et al., 2014). Also, another line of evidence demonstrated that intra-thalamic microinjection of minocycline was sufficient to reverse sciatic nerve injury-induced thermal hyperalgesia for 30 min (LeBlanc et al., 2011). However, in an animal model of the spinal cord injury-induced mechanical pain hypersensitivity, it was shown that intra-thalamic minocycline microinjection produced anti-hyperalgesic effect for 8 h (Zhao et al., 2007). Herein, our results showed the duration of the analgesic effect of minocycline on CPSP was much longer and lasted at least for 7 days. The discrepancy in duration of analgesia for minocycline might be duo to various reasons such as differences in the time course of microglial cell activation in different pain models, drug delivery route and other unknown mechanisms involved. Unlike the sciatic nerve and spinal cord injury, thalamic hemorrhage was the primary injury in CPSP model and glial activation in the thalamus would be the critical underlying mechanism for CPSP which was the reason for us to adopt intra-thalamic injection in the present study. It has been already shown that the activated glial cells of both microglia and astrocytes can produce and release various types of chemokines and cytokines that not only trigger neuronal hyperexcitability but also facilitate inter-glial cell activations through positive feedback regulation (Guo et al., 2007; Sheridan and Murphy, 2013; Macht, 2016). Here we further demonstrated that chemokine SDF1 and its cognate receptor CXCR4 were both up-regulated in the peri-thalamic lesion sites after ITC and CXCR4 was localized not only in neurons but also in astrocytes and microglia. In line with previous studies, the temporal pattern of SDF1 up-regulation was similar to the up-regulation of GFAP and Iba1, suggesting that activated microglia and astrocytes are the main SDF1-producing cells in the peri-thalamic lesion sites (Shen et al., 2014; Bai et al., 2016; Yang L. et al., 2016). Based on the cellular localization of CXCR4 in both neurons and glial cells and the abundant production of SDF1 from activated astrocytes and microglia, we propose that through positive feedback regulation, SDF1-CXCR4 signaling would play critical roles in mediating microglial-astrocytic-neuronal interactions, because selectively blocking CXCR4 with intra-thalamic AMD3100 microinjection could: (1) remarkably suppress thalamic hemorrhage-induced microglia and astrocyte activation; (2) significantly inhibit the release of pro-inflammatory mediators such as TNF-α, IL-1β and IL-6 from activated glial cells; and (3) reverse thalamic hemorrhage-induced increase in neuronal activities labeled with c-Fos in the peri-thalamic lesion sites. The persistent release of pro-inflammatory mediators could cause neuronal hyperexcitability and sensitization through binding to their respective receptors (Zhang et al., 2011; Clark et al., 2013; Chirila et al., 2014). As a new line of evidence, we have previously demonstrated that blocking CXCR4 with AMD3100 directly inhibited the hyperexcitability of primary nociceptor neurons under peripheral inflammatory pain state (Yang et al., 2015). Thus, it is conceivable that SDF1-CXCR4 signaling could mediate microglial-astrocytic-neuronal interactions (crosstalk) through two possible mechanisms: (1) glial-neuronal interactions mediated by direct binding of astrocyte/microglia-derived SDF1 with neuronal CXCR4; and (2) positive feedback glial-glial interactions through direct binding of astrocyte- or microglia-derived SDF1 with glial CXCR4 (Figure 10). The glial-glial interactions may serve as the source of pro-inflammatory mediators that in turn cause neuronal hyperexcitability and sensitization.

Given the potential role of SDF1-CXCR4 signaling in the glial-glial interactions and glial-neuronal crosstalk, we here demonstrated that pre-treatment with CXCR4 inhibitor AMD3100 could prevent the development of CPSP accompanying with the reduction of neuroinflammation which was essential for CPSP. To further confirm whether SDF1-CXCR4 signaling participates in the CPSP process, we injected exogenous SDF1 into VPL of naïve rats. As expected, single intra-thalamic SDF1 injection was sufficient to induce bilateral mechanical pain hypersensitivity through CXCR4 agonism. In addition, the SDF1-induced pain hypersensitivity was prevented by inhibiting microglial and astrocytic activation. It was interesting to note that both the analgesic effect of intra-thalamic AMD3100 and hyperalgesic effect of intra-thalamic SDF1 remained for a relative longer duration after administration although AMD3100 and SDF1 themselves have a short-term half-life (Hendrix et al., 2004; Kirkpatrick et al., 2010). Why? One possibility is that SDF1-CXCR4 signaling serves as a priming signal to mediate a positive feedback between glial-glial interactions under CPSP state. Once being activated, the downstream inflammatory cascades (TNF-α, IL-1β and IL-6) would be recruited to promote the local persistent neuroinflammation in the lesioned thalamus (Figure 10). This was supported by a previous study showing that intra-thalamic injection of CCL21 induced significant microglia activation and pain hypersensitivity which lasted longer than its own half-life and intra-thalamic injection of minocycline could also suppress CCL21-induced changes (Zhao et al., 2007). Moreover, here we also showed that post-treatment with AMD3100 could reverse the established CPSP in a dose-related manner. Taken all the existing data into account, we conclude that SDF1-CXCR4 signaling-mediated glial-glial/glial-neuronal communications are required for the development and maintenance of CPSP. Since it has been demonstrated that treatment with P2X7 receptor antagonist and IL-1β antibody could also reduce thalamic hemorrhage-induced microglia aggregation, neuronal excitability and allodynia phenomena in CPSP rats (Kuan et al., 2015), a common upstream regulation by HIF-1α-SDF1-CXCR4 signaling might be shared.

Because the analgesic effect of intra-thalamic injection of lidocaine on the thalamic hemorrhage-induced tactile hypersensitivity is short-lasting (Gritsch et al., 2016), the use of AMD3100 (also named as plerixafor) to treat CPSP would be more promising. Its safety and efficacy was approved by the U.S. Food and Drug Administration in 2008 for the mobilization of hematopoietic stem cells (Hummel et al., 2014). Moreover, several other CXCR4 antagonists are currently in clinical trials for cancer, HIV and WHIM syndrome treatment, highly supporting its potential use for the treatment of CPSP in clinic (Debnath et al., 2013). Since intraperitoneal delivery of AMD3100 has been demonstrated to be effective in suppression of pain (Dubovy et al., 2010; Xie et al., 2016), SDF1-CXCR4 signaling pathway as a new therapeutic target for the treatment of CPSP might be promising.

## Author Contributions

JC and FeiY conceived and designed the experiments. FanY, W-JL, WS, YW, C-LL and NW performed the experiments, FeiY and JC analyzed and interpreted the data. J-LW, FanY and X-LW supplied SPF animals. JC, S-MG and FeiY wrote the article. All authors listed, have made substantial, direct and intellectual contribution to the work, and approved it for publication.

## Conflict of Interest Statement

The authors declare that the research was conducted in the absence of any commercial or financial relationships that could be construed as a potential conflict of interest.
